# Suicidal Ideation Among Adolescents—The Role of Sexual Abuse, Depression, and Impulsive Behavior

**DOI:** 10.3389/fpsyt.2021.726039

**Published:** 2021-12-20

**Authors:** Pradeep Kumar, Shobhit Srivastava, Prem Shankar Mishra, Debashree Sinha

**Affiliations:** ^1^Department of Mathematical Demography & Statistics, International Institute for Population Sciences (IIPS), Mumbai, India; ^2^Department of Population Research Centre, Institute for Social and Economic Change, Bengaluru, India

**Keywords:** suicidal ideation, adolescents, gender, depression, sexual abuse, impulsive behavior

## Abstract

Suicide is the second leading cause of death among adolescents. With every fifth Indian to be an adolescent, the cost of an adolescent dying by suicide is enormous. This necessitates an understanding of the potential risk factors of suicidal ideation among adolescents. Secondary data analysis is performed on cross-sectional survey data obtained from Understanding the Lives of Adolescents and Young Adults. The survey was conducted in two Indian states of Bihar and Uttar Pradesh. Descriptive statistics, bivariate analysis and logistic regression are done to examine the results. Adolescent girls reported more suicidal ideation than boys. The odds of suicidal ideation are significantly higher among adolescents whose mother faced physical abuse and adolescents who themselves experienced sexual abuse. Adolescent boys and girls who have moderate depressive symptoms and impulsive behavior are significantly more likely to report suicidal ideation. The results help in identifying the adolescents who are at a particular risk for suicidal ideation while planning for intervention program for prevention of suicide.

## Introduction

Across the world in the year 2014, an estimated 804,000 deaths by suicide was reported by the World Health Organization. As per the report, the rate of suicide was 11.4 per 100,000 populations (1). Although the rate of suicide is observed to be higher in the high-income countries, a high burden of suicide in absolute term (total cases) is observed in low-and-middle-income countries as well. With an estimate of 75% of all suicides that occur in low and middle-income countries (1, 2) Causes of Death Collaborators, 2017), one cannot neglect the severity of the situation. Despite the continuous advancement in neuroscience as well as in the knowledge of human behaviors pathophysiology, currently suicidal behavior represents a puzzling challenge (3), and it has got little empirical attention worldwide (4). Furthermore, together with underreported suicide deaths and lack of availability of quality data it has become a major challenge for formulating an effective suicide prevention program in these countries (1, 5–9). Again, although the Member States of the WHO Mental Health Action Plan 2013–2020 committed to work toward the global target of reducing the suicide rate in countries by 10% by 2020 (1), lack of timely and effective evidence-based interventions, treatment, and support for both suicides and suicidal attempts were seen as major obstacles in achieving the above-mentioned targets in the low and middle-income countries (8, 10–13).

The greater cause of suicidal ideation among adolescents has been observed from the genetics and family environment that results as mood disorders, impulsive and aggressive behavior and these are the adoptive behaviors rather than biological in nature (14, 15). However, it is unknown which one is dominating in nature. Furthermore, substance abuse, sexual abuse, psychological distress, and physical inactivity among adolescents might be also responsible and play an intertwined role in creating an environment for suicidal ideation and it is well-linked to this age group (16). Familial, peer group and community negativity has led to low-self image among them (14, 17) In this way, it is a complex phenomenon especially among adolescents to understand suicidal ideation while correlating with the risk predictors.

In India, as per Census 2011, the youth population (15–24 years) is 231.9 million and that of the adolescent (10–19 years) is 253.2 million, constituting 19.2 and 20.9% of the total population, respectively (18). According to the WHO report, suicide is the second leading cause of death among young people aged 15–29 years (1). Unfortunately, the increasing trend of suicidal ideation among youth and adolescents is continuing to remain high in India and across its states (2, 19, 20). Therefore, it has become a serious social and public health issue. Every year, more than 100,000 people die by suicide in India (20, 21). The rates of suicide in India have increased from 9.9% in 2017 to 10.2% in 2018, and the share of adolescent suicide to the total suicide is ~7% in the same period (20).

Self-harm or self-destruction is a major risk factor for dying by suicide that has substantially risen over the period (22–24). Dying by suicide is a multi-casual relation (25) that can be understood through the interpersonal theory of suicide (4). Furthermore, suicidal ideation among adolescents is driven by multiple risk factors like psychiatric disorders, mood disorders, anxiety, mental disorders, substance abuse, personality disorders, and other co-morbidities (26, 27). The other risk factors that have been identified for suicidal ideation among adolescents (below 18 years) are family problems, failure in examination, love affairs, and illness (social and mental problems) (20, 28). For instance, family problems (30.4%) and illness (17.7%) have together contributed 48% of total suicides in India (20). Again, both mental illness and social factors are highly linked to suicide or suicidal ideation among adolescents and youth populations (14, 26, 29). Previous studies found that there is a significant correlation between predictors of suicide such as male gender, increasing age, previous suicide attempts, alcohol and substance abuse, and ongoing or previous mental and psychiatric treatment (2, 6, 24, 30). Therefore, the present study tries to explore the possible risk factors that influence suicidal ideation among adolescent population of two of the biggest states in India, Uttar Pradesh and Bihar.

### Conceptual Framework

According to WHO, suicidal behavior is defined as a “range of behavior that includes thinking about suicide (suicidal ideation), planning for suicide, attempting suicide, and committing suicide” (1). Existing literature on suicidal ideation among adolescents relies on a broad range of determinants from professional or career problems to sense of isolation, abuse, and violence, from family problems to mental and psychological disorders, addiction to alcohol, and financial loss (7, 8, 11, 13, 16, 31–33).

**Figure 1:**
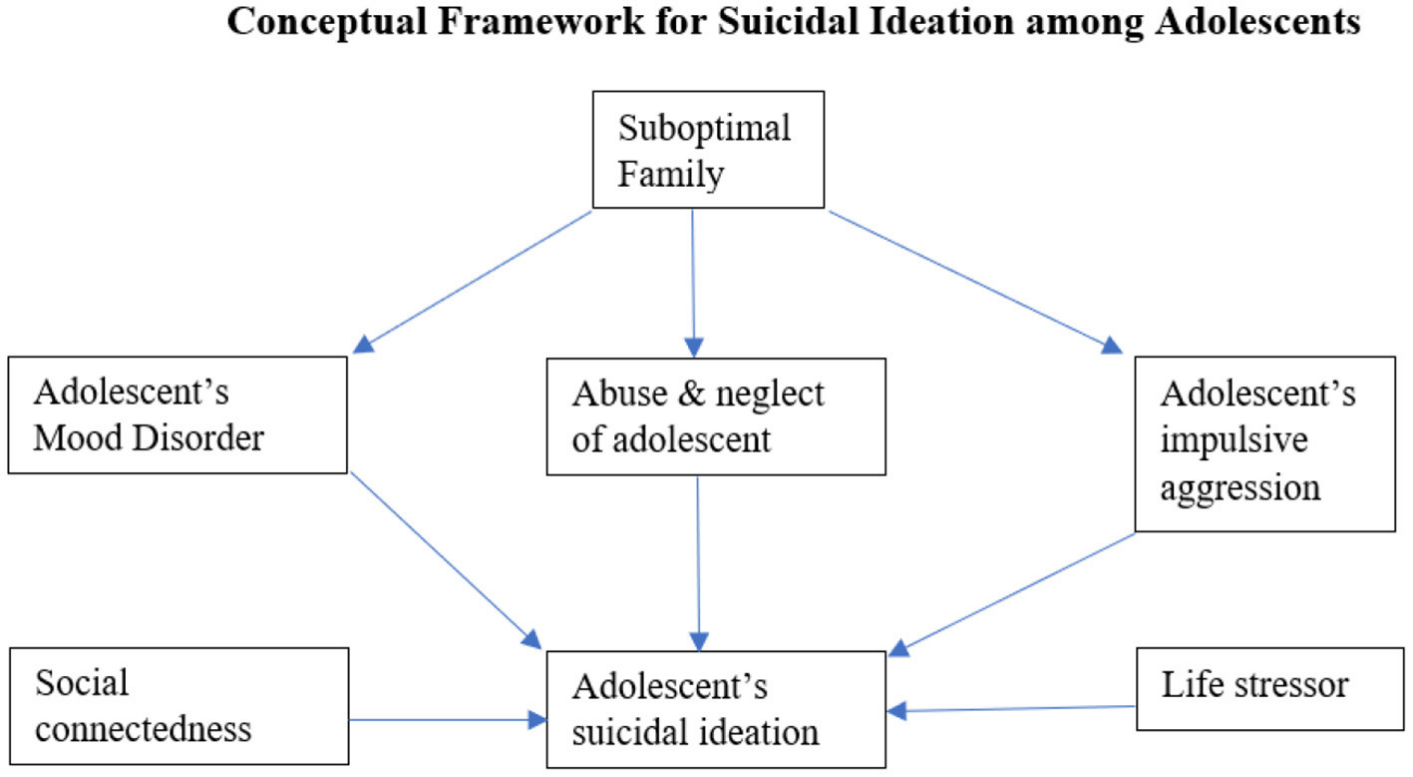
Conceptual framework for suicidal ideation among adolescents.

Given the broad range of determinants that are associated with suicidal ideation, the present research adapts the framework of “Familial Pathways to Early-Onset Suicidal Behavior,” given by Brent and Mann (25). The framework tries to explain that suicidal behavior that begins before the age of 25 is highly familial. The authors propose a stress-diathesis model along with familial transmission of vulnerabilities to suicidal behavior. Although with the available data, we had to exclude some of the variables proposed in the original framework (i.e., parent's suicide attempt, parent's mood disorder, parent's impulsive aggression) we tried to include another relevant variable (i.e., social connectedness) which have not yet been explored in the context of suicidal ideation among adolescents. Figure 1 given below illustrates how suboptimal family can lead to mood disorder, impulsive aggression, and abuse and neglect of an adolescent which ultimately leads to his/her suicide attempt. Further, social connectedness (communication with parents, having friends, usage of social media) and life stressors (class performance, residence before migration, freedom to move etc.) can also act as risk factors that lead to suicide ideation.

### Purpose of the Paper

Suicidal ideation is an impulsive behavior that emerges often in the adolescent population (4, 34, 35). A systematic review on suicidal ideation found that the existing knowledge on suicidal ideation among adolescents is still not well-understood and well-established. For example, there is no specific cause for suicidal ideation (1, 14, 26, 29, 36–40). In addition, adolescent suicide varies according to various socioeconomic levels, gender and age-groups (21, 28, 38, 41). Therefore, an assessment of the socio-economic and psychological health status is needed for a comprehensive understanding of suicidal behavior among adolescents.

In India, every fifth person is an adolescent (10–19 years) (18). Recent literature suggests that suicide among them is increasing (8, 42). Moreover, Uttar Pradesh being the most populous state in the country accounts for the largest number of adolescent (48.9 million) and youth (40.6 million) population; that accounts for 19.3 and 17.5% of the total adolescent and youth population of the country, respectively (18). Again, Bihar is the second state that contributes to the highest adolescent (9.2%) and youth population (7.6%) in the country's total population. With no national suicide prevention strategy in India, it becomes important to study the suicidal ideation among adolescents in two of the biggest states in India. Therefore, the study aims to understand and assess multiple precipitating risk factors for suicidal ideation among adolescents.

## Materials and Methods

### Material

Secondary data analysis is performed on cross-sectional survey data obtained from Understanding the Lives of Adolescents and Young Adults (UDAYA) project survey. The survey was conducted in two Indian states of Uttar Pradesh and Bihar, in 2016 by Population Council under the guidance of Ministry of Health and Family Welfare, Government of India. The UDAYA survey collected detailed information on family, media, community, environment, assets acquired in adolescence, and quality of transitions from young to adulthood indicators. The sample size for Uttar Pradesh and Bihar was 10,350 and 10,350 adolescents aged 10–19 years, respectively. UDAYA was designed to provide estimates for the state as a whole as well as for the urban and rural areas of the state for each of the five categories of respondents, namely younger boys in ages 10–14 years, older boys in ages 15–19 years, younger girls in ages 10–14 years, unmarried older girls in ages 15–19 years, and married older girls in ages 15–19 years. The required sample for each sub-group of adolescents was determined at 920 younger boys (11–14 years), 2,350 older boys (15–19 years), 630 younger girls (11–14 years), 3,750 older girls (15–19 years), and 2,700 married girls in both states. The study treated rural and urban areas of the state as independent sampling domains and therefore, drew sample areas independently for each of these two domains. The 150 Primary Sampling Units (PSUs) were further divided equally into rural and urban areas, i.e., 75 for rural respondents and 75 for urban respondents. Within each sampling domain, the study adopted a multi-stage systematic sampling design. The 2011 census list of villages and wards [each consisting of several census enumeration blocks (CEBs) of 100–200 households] served as the sampling frame for the selection of villages and wards in rural and urban areas, respectively. This list was stratified using four variables, namely, region, village/ward size, proportion of the population belonging to scheduled castes and scheduled tribes, and female literacy.

The household sample in rural areas was selected in three stages, while in urban areas it was selected in four stages. In rural areas, villages were first selected systematically from the stratified list as described above, with selection probability proportional to size (PPS). In urban areas, 75 wards were first selected systematically with probability proportional to size, and within each selected ward, CEBs were then arranged by their administrative number and one CEB was randomly selected. Several CEBs adjacent to the selected CEB were merged to ensure at least 500 households for listing. The detailed about sampling design and survey methodology is published elsewhere (43). The effective sample size for this study was 4,667 and 8,465 adolescent boys and girls aged 13–19 years. The age group is taken as 13–19 years as the question regarding suicidal ideation was asked to this age group only.

### Variable Description

The variables used in the study are presented in Table 1.

**Table 1:** Description of variables used in the study.

***Outcome variable***		
**Suicidal ideation**	During the past 1 year, did you ever seriously consider attempting suicide?	No and Yes
***Explanatory variables***		
**Social variables**		
Education	Are you currently studying (regular and/or correspondence)?	No and Yes
Media exposure	How often do you watch television or radio or read newspaper?	No, rarely and frequently
**Abuse and neglect of the adolescent**		
Physical abuse with mother	Has your father beaten your mother in the last 12 months?	No and Yes
Physical abuse with respondent	Have you been physically hurt (for example, beaten) by your father or mother from the time you turned 10 years old?	No and Yes
Sexual abuse with respondent	Has anyone ever touched you in a bad way when you didn't want to be touched, for example your private parts?	No and Yes
**Social connectedness**		
Communication with parents	Do you feel that you can talk about personal things with parents and they will listen?	Difficult and easy
Respondent have friends	Does the respondent have any friend?	No and Yes
Use of social media	How often do you use Facebook, twitter or other social media?	Not at all, rarely and frequently
**Life stressor**		
Class performance	In the last academic year, how did you perform in your class?	Not attending school, poor/average and good
Residence before migration	Just before you moved here, did you live in a city, in a town or in a village?	No migration, city/town and village
Freedom to move	Are you usually allowed to go alone to a shop or market or visit a friend/relative inside your village/ward or outside your village/ward or attend any programme (a mela, sports event, girls' group meetings) inside your village/ward?	No and Yes
Expression of opinion (in family)	Do you express your opinion to elders in your family?	No and Yes
Confront wrong act	If someone says or does something wrong to you, do you confront that person or just stay quiet?	No and Yes
Engagement or marriage decided	Are you engaged or has your marriage been fixed?	No and Yes
**Sub-optimal family environment**		
Any adult as a role model	Is/are there any adult(s) who you see as a role model?	No and Yes
Decision about up to which class “r” would study	Who mainly decides till which standard you will study?	Alone and with others/no schooling
Discuss about gynecological problems	Suppose you have a problem in your private parts such as itching or burning while passing urine or pain during menstruation, who are you most likely to talk to about this?	No if she talks to no one and yes if she talks to anyone in the family
Discussion about relationships (girl/boy)	Who are you most likely to talk to about your relationship with a male friend or a boyfriend?	No if she talks to no one and yes if she talks to anyone in the family
**Adolescent's mood disorder**		
Depressive symptoms	The questions included, a. had trouble falling asleep or sleeping too much, b. feeling tired or having little energy, c. poor appetite or eating too much, d. trouble concentrating on things, e. had little interest or pleasure in doing things f. feeling down, depressed or hopeless, g. feeling bad about yourself, h. been moving or speaking slowly, i. had thoughts that respondent would be better off dead (Cronabach Alpha: 0.89)	Minimal/Mild, Moderate and Moderately high/severe (43)
**Adolescent's impulsive/aggressive behavior**		
Impulsive behaviors	During the past 12 months, when you are agitated, angry or sad, have you ever cut/bitten yourself or have you ever pulled your own hair or have you ever banged or hit yourself	No and yes

*Proportion test is used to check the significant difference of suicidal ideation between adolescent boys and girls*.

### Statistical Analysis

Descriptive statistics along with bivariate analysis is done to examine the preliminary results. For analyzing the association between the binary outcome variable (suicidal ideation) and other explanatory variables binary logistic regression method is used. The outcome variable is suicidal ideation among adolescents aged 13–19 years. The explanatory variables were grouped as social variables, abuse and neglect of the adolescent, social connectedness, life stressor, sub-optimal family environment, adolescent's mood disorder, adolescent's impulsive/aggressive behavior. The multivariable regression analysis is used to determine the association between the outcome and explanatory variables.

### Ethical Considerations

The ethical approval for this data was provided by the Population Council Institutional Review Board. Adolescents provided individual written consent to participate in the study, along with a parent/guardian for adolescents younger than 18. The Population Council identified referral services for counseling and health services to offer respondents if necessary, and fieldworkers were trained on ethical issues and sensitivity. In addition, interviewing boys and girls in separate segments helped minimize issues related to confidentiality and response bias (44).

## Results

### Socio-Demographic Profile of Adolescents Aged 13–19 Years by Gender

Table 2 shows that majority of adolescents (both boys and girls) were educated and frequently used mass media. About 55 per cent adolescent boys and 32 per cent of girls experienced physical abuse; nearly two per cent boys and seven per cent girls reported sexual abuse. Around 79 per cent respondents (both boys and girls) had social behavior. About 16 per cent of adolescent boys use social media frequently, whereas 3 per cent of adolescent girls use social media frequently. Nearly 30 per cent adolescents (both boys and girls) had good class performance, and adolescent boys (99%) had more freedom to move compared to girls (74.6%). The majority of adolescents (both boys and girls) had the expression of opinions in their family, moreover, girls (80.2%) confronted more wrong acts than boys (74.5%). About 43 per cent of boy and 35 per cent of girl respondents had any adults as role model, and boys alone (49.5%) made more decision about which class they would study than girls (23.6%). Adolescent girls (79.2%) discussed more about their relationship with boy/girl compared to boys (76.9%). Similarly, girls reported higher moderate depressive symptoms than boys. Impulsive behavior was higher among boy respondents (4.1%) compared to girls (2.6%).

**Table 2:** Socio-demographic profile of adolescents aged 13–19 years.

**Variables**	**Adolescent boys**		**Adolescent girls**	
	**Sample**	**Percentage**	**Sample**	**Percentage**
**SOCIAL VARIABLES**				
**Schooling status**				
Never/attended/dropout	1,216	26.1	3,120	36.9
Currently attending	3,451	74.0	5,345	63.1
**Media exposure**				
No	236	5.1	1,205	14.2
Rarely	775	16.6	2,279	26.9
Frequently	3,656	78.3	4,981	58.9
**ABUSE AND NEGLECT**				
**Physical abuse with mother**				
No	4,410	94.5	7,804	92.2
Yes	257	5.5	661	7.8
**Physical abuse with respondent**				
No	2,099	45.0	5,721	67.6
Yes	2,568	55.0	2,744	32.4
**Sexual abuse with respondent**				
No	4,588	98.3	7,836	92.6
Yes	79	1.7	629	7.4
**SOCIAL CONNECTEDNESS**				
**Communication with parents**				
Difficult	976	20.9	1,739	20.6
Easy	3,691	79.1	6,726	79.5
**Respondent have friends**				
No	128	2.7	419	5.0
Yes	4,539	97.3	8,046	95.1
**Use of social media**				
Not at all	3,616	77.5	8,044	95.0
Rarely	304	6.5	169	2.0
Frequently	747	16.0	252	3.0
**LIFE STRESSOR**				
**Class performance**				
Not attending school	1,344	28.8	3,545	41.9
Poor/average	2,021	43.3	2,464	29.1
Good	1,302	27.9	2,456	29.0
**Residence before migration**				
No migration	3,964	84.9	7,510	88.7
City/town	463	9.9	475	5.6
Village	240	5.1	481	5.7
**Freedom to move**				
No	39	0.8	2,147	25.4
Yes	4,628	99.2	6,318	74.6
**Expression of opinions (in family)**				
No	573	12.3	1,297	15.3
Yes	4,094	87.7	7,168	84.7
**Confront wrong acts**				
No	1,190	25.5	1,675	19.8
Yes	3,477	74.5	6,790	80.2
**Engaged or marriage decided**				
No	4,636	99.3	8,130	96.0
Yes	31	0.7	335	4.0
**SUB-OPTIMAL FAMILY ENVIRONMENT**				
**Any adults as a role model?**				
No	2,650	56.8	5,468	64.6
Yes	2,017	43.2	2,997	35.4
**Decision about up to which class “r” would study**				
Alone	2,312	49.5	1,997	23.6
With others/no schooling	2,355	50.5	6,468	76.4
**Discuss about gynecological problems**				
No	184	3.9	293	3.5
Yes	4,483	96.1	8,172	96.5
**Discussion about relationships (girl/boy)**				
No	1,078	23.1	1,760	20.8
Yes	3,589	76.9	6,705	79.2
**MOOD DISORDER**				
**Depressive symptoms**				
Minimal/mild	4,597	98.5	8,077	95.4
Moderate	54	1.2	261	3.1
Moderately high/severe	16	0.4	127	1.5
**IMPULSIVE/AGGRESSIVE BEHAVIOR**				
**Impulsive behavior**				
No	4,478	95.9	8,249	97.4
Yes	189	4.1	216	2.6
**Total**	4,667	100	8,465	100.0

### Bivariate Associations of Suicidal Ideation With Adolescent's Background Characteristics by Gender

The percentage distribution of suicidal ideation among adolescents aged 13–19 years by background characteristics is presented in Table 3. Overall, adolescent girls (4.2%) reported more suicidal ideation than boys (2.1%). Suicidal ideation was significantly higher among adolescent girls (4.8%) and boys (3.2%) who never attended/dropped out of school than those who were currently attending school (*p* < 0.001). Adolescents who had frequent media exposure reported significantly higher prevalence of suicidal ideation (girls-4.5% and boys-2.4%) (*p* < 0.001).

**Table 3:** Percentage distribution of suicidal ideation among adolescents aged 13–19 years.

**Variables**	**Adolescent boys**	**Adolescent girls**	**Difference (*p*-value)**	
	**%**	**%**		**Cohen's d (C.I.)**
**Social variables**				
**Schooling status**				
Never/attended/dropout	3.2	4.8	−1.7 (0.001)	−0.08 (−0.15, −0.01)
Currently attending	1.8	3.8	−2.0 (0.001)	−0.13 (−0.17, −0.08)
**Media exposure**				
No	0.7	3.7	−3.1 (0.001)	−0.14 (−0.30, 0.02)
Rarely	1.3	3.6	−2.3 (0.001)	−0.15 (−0.24, −0.06)
Frequently	2.4	4.5	−2.1 (0.001)	−0.11 (−0.15, −0.07)
**ABUSE AND NEGLECT**				
**Physical abuse with mother**				
No	1.7	3.8	−2.0 (0.001)	−0.11 (−0.15, −0.08)
Yes	9.1	8.7	0.4 (0.984)	−0.05 (−0.20, 0.11)
**Physical abuse with respondent**				
No	1.5	3.3	−1.8 (0.001)	−0.12 (−0.16, −0.07)
Yes	2.7	6.0	−3.3 (0.001)	−0.17 (−0.22, −0.11)
**Sexual abuse with respondent**				
No	1.8	3.7	−2.0 (0.001)	−0.11 (−0.14, −0.07)
Yes	25.0	9.3	15.7 (0.001)	0.35 (0.10, 0.60)
**SOCIAL CONNECTEDNESS**				
**Communication with parents**				
Difficult	3.8	6.6	−2.8 (0.001)	−0.18 (−0.26, −0.10)
Easy	1.7	3.5	−1.8 (0.001)	−0.10 (−0.14, −0.06)
**Respondent have friends**				
No	1.7	3.6	−1.9 (0.001)	−0.13 (−0.33, 0.08)
Yes	2.2	4.2	−2.0 (0.001)	−0.12 (−0.15, −0.08)
**Use of social media**				
Not at all	1.7	4.1	−2.4 (0.001)	−0.13 (−0.17, −0.09)
Rarely	3.2	4.4	−1.3 (0.001)	−0.09 (−0.24, 0.07)
Frequently	4.0	5.1	−1.1 (0.001)	−0.17 (−0.28, −0.06)
**LIFE STRESSOR**				
**Class performance**				
Not attending school	3.1	4.8	−1.7 (0.001)	−0.09 (−0.15, −0.02)
Poor/average	1.8	4.0	−2.2 (0.001)	−0.12 (−0.17, −0.06)
Good	1.6	3.4	−1.8 (0.001)	−0.14 (−0.20, −0.07)
**Residence before migration**				
No migration	2.1	3.9	−1.8 (0.001)	−0.11 (−0.15, −0.07)
City/town	2.8	7.2	−4.3 (0.001)	−0.17 (−0.29, −0.05)
Village	1.9	4.7	−2.8 (0.001)	−0.12 (−0.26, 0.01)
**Freedom to move**				
No	0.0	3.3	−3.3 (0.001)	−0.20 (−0.49, 0.10)
Yes	2.2	4.5	−2.3 (0.001)	−0.12 (−0.16, −0.08)
**Expression of opinions (in family)**				
No	1.9	6.7	−4.8 (0.001)	−0.23 (−0.33, −0.12)
Yes	2.2	3.7	−1.5 (0.001)	−0.09 (−0.13, −0.06)
**Confront wrong acts**				
No	0.9	3.4	−2.6 (0.001)	−0.16 (−0.23, −0.08)
Yes	2.6	4.3	−1.7 (0.001)	−0.10 (−0.14, −0.06)
**Engaged or marriage decided**				
No	2.2	4.0	−1.9 (0.001)	−0.11 (−0.15, −0.07)
Yes	0.0	6.7	−6.7 (0.001)	−0.28 (−0.69, 0.13)
**SUB-OPTIMAL FAMILY ENVIRONMENT**				
**Any adults as a role model?**				
No	1.4	3.6	−2.2 (0.001)	−0.12 (−0.17, −0.07)
Yes	3.1	5.2	−2.1 (0.001)	−0.12 (−0.18, −0.07)
**Decision about up to which class “r” would study**				
Alone	2.6	3.0	−0.4 (0.324)	−0.07 (−0.13, −0.01)
With others/no schooling	1.7	4.5	−2.8 (0.001)	−0.14 (−0.18, −0.09)
**Discuss about gynecological problems**				
No	0.1	4.3	−4.2 (0.001)	−0.23 (−0.43, −0.03)
Yes	2.2	4.1	−1.9 (0.001)	−0.11 (−0.15, (−0.08)
**Discussion about relationships (girl/boy)**				
No	1.0	3.5	−2.5 (0.001)	−0.12 (−0.21, −0.04)
Yes	2.5	4.3	−1.8 (0.001)	−0.11 (−0.15, −0.07)
**MOOD DISORDER**				
**Depressive symptoms**				
Minimal/mild	2.1	3.3	−1.3 (0.001)	−0.07 (−0.11, −0.04)
Moderate	8.3	19.8	−11.5 (0.001)	−0.25 (−0.55, 0.05)
Moderately high/severe	1.8	24.7	−22.9 (0.001)	−0.48 (−1.01, 0.05)
**IMPULSIVE/AGGRESSIVE BEHAVIOR**				
**Impulsive behavior**				
No	1.5	3.7	−2.2 (0.001)	−0.12 (−0.15, −0.08)
Yes	16.9	21.7	−4.8 (0.001)	−0.30 (−0.49, −0.11)
**Total (** ***N*** **)**	2.1 (93)	4.2 (345)	−2.0 (0.001)	

*%, Percentage; C.I., confidence interval; Difference: Adolescent boys - Adolescent girls. p-value based on proportion test*.

The suicidal ideation was significantly higher among adolescents whose mothers experienced physical abuse (girls-8.7% and boys-9.1%) (*p* < 0.001). Similarly, adolescents who faced physical (girls-6% and boys-2.7%) or sexual abuse (girls-9.3% and boys-25%) reported significantly more suicidal ideation compared to those adolescents who did not face either of the abuse (*p* < 0.001). Moreover, adolescents who had no ease of communication with parents reported higher prevalence of suicidal ideation (girls-6.6% and boys-3.8%). Adolescent girls (5.1%) and boys (4%) who used social media frequently reported significantly higher suicidal ideation than those who did not used social media at all (*p* < 0.001). Class performance and residence before the migration of adolescents had a significant association with suicidal ideation. Girls who could not express their opinions in family reported significantly higher prevalence of suicidal ideation (6.7%) (*p* < 0.001). The suicidal ideation was significantly more among adolescents (girls-4.3% and boys-2.6%) who had to confront wrong acts than who did not. The prevalence of suicidal ideation was higher among adolescent girls who were engaged or whose marriage was decided (6.7%) (*p* < 0.001).

On the other hand, adolescents who had any adults as a role model (girls-5.2% and boys-3.1%) reported a higher prevalence of suicidal ideation than who did not. Suicidal ideation was significantly more prevalent among adolescent girls whose decision about which class they would study was taken by others/no schooling (4.5%) (*p* < 0.001). Adolescent girls (4.3%) and boys (2.5%) who discussed about their relationship with boys/girl had higher suicidal ideation.

Girls (24.7%) and boys (8.3%) who had moderately high/severe depressive symptoms (24.4%) reported a significantly higher prevalence of suicidal ideation than those who had minimal/mild symptoms. Adolescent impulsive behavior had a significant effect on the suicidal ideation. For instance, adolescent girls (21.7%) and boys (16.9%) who had impulsive behavior reported more suicidal ideation.

### Multivariate Associations of Suicidal Ideation With Adolescent's Background Characteristics by Gender

Table 4 provides the estimates from logistic regression analysis for suicidal ideation among adolescents aged 13–19 years. Though not significant, the result revealed that the likelihood of suicidal ideation was higher among adolescents who had frequent mass media exposure compared to those who did not have media exposure. The odds of suicidal ideation were significantly higher among adolescents whose mother experienced physical abuse (for boys–OR: 3.79; CI: 2.02–7.12 and girls-OR: 1.96; CI: 1.4–2.76), or adolescents who themselves faced sexual abuse (for boys-OR: 7.12; CI: 3.44–14.72 and girls-OR: 2.09; CI: 1.54–2.82) compared to their counterparts.

**Table 4:** Estimates from logistic regression analysis for suicidal ideation among adolescents aged 13–19 years.

**Variables**	**Adolescent boys**	**Adolescent girls**
	**OR (95% CI)**	**OR (95% CI)**
**Social variables**		
**Schooling status**		
Never/attended/dropout	Ref.	Ref.
Currently attending	0.48 (0.11,2.06)	1.09 (0.65,1.84)
**Media exposure**		
No	Ref.	Ref.
Rarely	1.51 (0.27,8.43)	1.11 (0.7,1.75)
Frequently	2.42 (0.5,11.76)	1.07 (0.7,1.63)
**Abuse and neglect**		
**Physical abuse with mother**		
No	Ref.	Ref.
Yes	3.79^*^(2.02,7.12)	1.96^*^(1.4,2.76)
**Physical abuse with respondent**		
No	Ref.	Ref.
Yes	1.51 (0.94,2.42)	1.24 (0.96,1.6)
**Sexual abuse with respondent**		
No	Ref.	Ref.
Yes	7.12^*^(3.44,14.72)	2.09^*^(1.54,2.82)
**Social connectedness**		
**Communication with parents**		
Difficult	Ref.	Ref.
Easy	0.52^*^(0.32,0.84)	0.54^*^(0.42,0.7)
**Respondent have friends**		
No	Ref.	Ref.
Yes	2.43 (0.23,25.62)	1.37 (0.72,2.6)
**Use of social media**		
Not at all	Ref.	Ref.
Rarely	2.33^*^(1.14,4.8)	1.04 (0.54,2.01)
Frequently	1.95^*^(1.15,3.29)	1.52 (0.97,2.37)
**Life stressor**		
**Class performance**		
Not attending school	Ref.	Ref.
Poor/average	0.87 (0.2,3.75)	0.70 (0.41,1.17)
Good	0.75 (0.17,3.38)	0.72 (0.43,1.22)
**Residence before migration**		
No migration	Ref.	Ref.
City/town	0.82 (0.43,1.6)	1.71^*^(1.16,2.51)
Village	0.93 (0.41,2.09)	1.09 (0.71,1.68)
**Freedom to move**		
No	#	Ref.
Yes	#	1.12 (0.84,1.49)
**Expression of opinions (in family)**		
No	Ref.	Ref.
Yes	1.15 (0.57,2.33)	0.55^*^(0.4,0.74)
**Confront wrong acts**		
No	Ref.	Ref.
Yes	1.92 (0.97,3.8)	1.33 (0.95,1.85)
**Engaged or marriage decided**		
No	#	Ref.
Yes	#	1.49 (0.93,2.4)
**Sub-optimal family environment**		
**Any adults as a role model?**		
No	Ref.	Ref.
Yes	1.28 (0.81,2.02)	1.18 (0.92,1.51)
**Decision about up to which class “r” would study**		
Alone	Ref.	Ref.
With others/no schooling	1.0 (0.63,1.57)	1.37^*^(1.03,1.84)
**Discuss about gynecological problems**		
No	Ref.	Ref.
Yes	3.13 (0.4,24.44)	0.97 (0.48,1.95)
**Discussion about relationships (girl/boy)**		
No	Ref.	Ref.
Yes	1.63 (0.79,3.36)	1.62^*^(1.14,2.31)
**Mood disorder**		
**Depressive symptoms**		
Minimal/mild	Ref.	Ref.
Moderate	2.85^*^(1.01,8.04)	6.17^*^(4.37,8.73)
Moderately high/severe	1.51 (0.14,16.17)	7.64^*^(5.04,11.6)
**Impulsive/aggressive behavior**		
**Impulsive behavior**		
No	Ref.	Ref.
Yes	7.38^*^(4.31,12.65)	6.0^*^(4.23,8.51)

* *if p < 0.05*;

*Ref, Reference; OR, Odds Ratio; CI, Confidence Interval*;

# *, omitted because of no sample available*.

Adolescent boys (OR: 0.52; CI: 0.32–0.84) and girls (OR: 0.54; CI: 0.42–0.70) were 48 and 46% less likely to report suicidal ideation if they had ease of communication with parents, compared to their counterpart, respectively. Moreover, boys who used social media rarely (OR: 2.33; CI: 1.14–4.8) or frequently (OR: 1.95; CI: 1.15–3.29) were 2.33 and 1.95 times significantly more likely to report suicidal ideation compared to those who did not use at all. The results were also similar for girls but insignificant.

Adolescents whose class performance were poor/average and good were less likely to have suicidal ideation than those who had not attended school. However, adolescents who confront wrong acts were more likely to report suicidal ideation compared to those who did not. However, the results were not significant. The likelihood of suicidal ideation was 62% significantly more likely among adolescent girls (OR: 1.62; CI: 1.14–2.31) who discussed about their relationships with boy/girl than those who did not consult with anyone. Adolescent boys (OR: 2.85; CI: 1.01–8.04) and girls (OR: 6.17; CI: 4.37–8.73) who had moderate depressive symptoms were 2.85 times and 6.17 times significantly more likely to report suicidal ideation, respectively, compared to those who had minimal/mild depressive symptoms. Similarly, the likelihood of suicidal ideation was 7.38 times and 6 times significantly more likely among boys (OR: 7.38; CI: 4.31–12.65) and girls (OR: 6.0; CI: 4.23–8.51) who had impulsive behavior, respectively, compared to those who did not have impulsive behavior.

## Discussion

Based on our conceptual framework and subsequent analysis, the key findings of the present study are (i) Suicidal ideation is more among adolescent girls than adolescent boys. (ii) Adolescent boys and girls who had the ease of communication with their parents are less likely to have suicidal ideation. (iii) Adolescents who themselves faced either physical or sexual abuse and whose mothers experienced physical abuse reported to have more suicidal ideation. (iv) Adolescents who suffered from high/severe depression and impulsive behavior had more suicidal ideation

In agreement with previous research, the study results indicate that suicidal ideation is more among adolescent girls than adolescent boys (17, 34, 45, 46). One of the reasons for such an observation might be because of depressive symptoms during the post-pubertal period (47). Further, adolescents who are exposed to mass media, and frequently used social media are more likely to have suicidal ideation. A study on young adults and adolescents revealed that the most cited source of suicide stories were newspapers, friends, relatives, internet news sites and social networking sites (48). Since it has been established that exposure to suicide predicts suicidal attempts and ideation (49), parents and elders at home should be vigilant about adolescent's exposure to suicide stories through social media.

The study results also emphasized the importance of communication about one's personal matters with one's parents. For instance, adolescent boys and girls who had the ease of communication with their parents are less likely to have suicidal ideation. One possible explanation for this might be that strong bond and connectedness between the adolescent and his or her parent leads to a healthy behavioral and emotional health (50). The same study showed that unhealthy weight control, substance use, suicide attempts, body-dissatisfaction, depression and low self-esteem were found among adolescents who valued their friends' opinions over their parents. Similarly, suicidal behavior was found to be less among Lithuanian adolescents who spent time with family and had ease of communication with parents (15). Thus, the role of parents in preventing an adolescent's suicidal ideation cannot be ignored.

Adolescents who themselves faced either physical or sexual abuse and whose mother's experienced physical abuse reported more suicidal ideation (33). A study on the psychosocial correlates of suicidal attempts among Nigerian youth found sexual abuse to be a significant predictor of suicidal behavior (33). Similarly, a history of sexual abuse was found to be a risk factor for suicide attempts (51). Coping up with sexual abuse can leave any young adolescent in distress who might resort to self-destructive behavior like attempting or contemplating suicide. Moreover, adolescents who suffered from high/severe depression and impulsive behavior had more suicidal ideation. The results were similar with other studies among adolescents (25, 31, 46, 52, 53). Depression among adolescents can be explained because of less disposable monthly allowance, more cigarette smoking, feelings of being neglected, being abused in school and having a lower monthly family income (54).

The present study also included variables like freedom of movement, expression of opinion and whether engagement or marriage was decided or not as life stressors in an adolescent's life. The study results indicated that suicidal ideation was more among adolescent girls whose marriage or engagement was decided and who could not express their opinions. Existing research found an association of early marriage with adverse mental health outcomes (55, 56) and since depression is one of the leading causes of suicide among adolescents, one can rely on this interconnection.

### Implications of the Study

Suicidal ideation is considered to be the first step on the pathway to suicide (57). Therefore, adolescent suicidal ideation should be treated as a social and public health challenge for the country. The findings of the present study will help parents, healthcare providers, teachers, and adolescents themselves to identify the key elements of suicidal ideation that will ultimately lead to its prevention. Adolescents who have difficulty in communication with parents, who are victims of physical and sexual abuse and who have depression and impulsive behavior face several negative consequences such as stigmatizing, and discrimination on multiple grounds. This leads to social isolation, hopelessness, low self-esteem, and ultimately to self-harm. Hence, to curb down the suicidal rates, strategies such as regular communication with parents and school authorities, reduction in stigma related to suicidal ideation, raising of public awareness, social and psychological educators, police, and other gatekeepers can be effective (21, 36, 40, 42, 58).

### Limitations of the Present Study

The present study is not without limitations. First, due to the cross-sectional nature of data, we could not examine any cause-and-effect relationship between the dependent and the independent variables mentioned in the study. Second, no fatal consequence due to suicide was examined. Third, the time frame of “1 year” given in the question “During the past 1 year, did you ever seriously consider attempting suicide?” (i.e., the outcome variable) is too vast to give an accurate response. Fourth, the analysis is based on self-reported response given by adolescents aged 13–19 years which can be influenced by their immediate surrounding like presence of an elderly or a parent. Finally, since the analysis is only on two states of India, Bihar and Uttar Pradesh, one needs to be cautious while generalizing the results for the entire country.

## Conclusion

Given the significance of the topic in recent times, the present study provides insights into the characteristics of adolescents who have suicidal ideation and highlights its linkages with sexual abuse, depression and impulsive behavior. The results of the present study reveal that suicidal ideation is preventable if adolescents suffering from abuse, depression, impulsive aggression can be identified. In this context, the role of parents is pivotal in monitoring their children. This study brings special attention to policymakers to design strategic plans to reduce the incidence of suicide among adolescents by focusing on various risk factors of suicidal ideation.

## Data Availability Statement

The data that support the findings of this study are available at: https://dataverse.harvard.edu/dataset.xhtml?persistentId=doi:10.7910/DVN/RRXQNT; but restrictions apply to the availability of these data, which were used under license for the current study, and so are not publicly available. Requests to access these datasets should be made using the form found at https://pcouncil.wufoo.com/forms/qk55ehv048g4b6/.

## Author Contributions

PK: application of statistical techniques to analyze study data, writing, and interpretation of results. SS: application of statistical techniques to analyze study data, writing, and interpretation of results. PM: writing the initial draft and literature review. DS: critical review, revision, and finalizing the manuscript. All authors contributed to the article and approved the submitted version.

## Funding

This paper was written using data collected as part of Population Council's UDAYA study, which is funded by the Bill and Melinda Gates Foundation and the David and Lucile Packard Foundation. No additional funds were received for the preparation of the paper.

## Conflict of Interest

The authors declare that the research was conducted in the absence of any commercial or financial relationships that could be construed as a potential conflict of interest.

## Publisher's Note

All claims expressed in this article are solely those of the authors and do not necessarily represent those of their affiliated organizations, or those of the publisher, the editors and the reviewers. Any product that may be evaluated in this article, or claim that may be made by its manufacturer, is not guaranteed or endorsed by the publisher.

## References

[1] WHO. Preventing suicide: A Global Imperative. Luxembourg: World Health Organization (2014).

[2] GBD 2016 Causes of Death Collaborators. Global, regional, and national age-sex specific mortality for 264 causes of death, 1980–2016: a systematic analysis for the Global Burden of Disease Study 2016. Lancet. (2017) 390:1151–210. 10.1016/S0140-6736(17)32152-9 28919116 PMC5605883

[3] DeBerardis D Fornaro M Valchera A Cavuto M Perna G Di Nicola M. Eradicating suicide at its roots: preclinical bases and clinical evidence of the efficacy of ketamine in the treatment of suicidal behaviors. Int J Mol Sci. (2018) 19:1–22. 10.3390/ijms19102888 30249029 PMC6213585

[4] Orden KA Van Witte TK Cukrowicz KC Braithwaite S Selby EA. The interpersonal theory of suicide. Psychol Rev. (2010) 117:575–600. 10.1037/a0018697 20438238 PMC3130348

[5] Gibbons RD Hendricks Brown C Hur K Marcus SM Bhaumik DK Erkens JA. Early evidence on the effects of regulators' suicidality warnings on SSRI prescriptions and suicide in children and adolescents. Am J Psychiatry. (2007) 128:524–30. 10.1176/appi.ajp.2007.07030454 17728420

[6] Harris TE Lennings CJ. Suicide and adolescence. Int J Offender Ther Comp Criminol. (1993) 37:263–70. 10.1177/0306624X9303700307

[7] Picazo-Zappino J. Suicide among children and adolescents: a review. Actas Esp Psiquiatr. (2014) 42:125–32. 24844812

[8] Patel V Ramasundarahettige C Vijayakumar L Thakur JS Gajalakshmi V Gururaj G. Suicide mortality in India: a nationally representative survey. Lancet. (2012) 379:2343–51. 10.1016/S0140-6736(12)60606-0 22726517 PMC4247159

[9] Spirito A Brown L Overholser L Fritz G. Attempted suicide in adolescence: a review and critique of the literature. Clin Psychol Rev. (1989) 9:335–63. 10.1016/0272-7358(89)90061-59459699

[10] Bachmann S. Epidemiology of suicide and the psychiatric perspective. Int J Environ Res Public Health. (2018) 15:1–23. 10.3390/ijerph15071425 29986446 PMC6068947

[11] Gould MS Greenberg T Velting DM Shaffer D. Youth suicide risk and preventive interventions: a review of the past 10 years. J Am Acad Child Adolesc Psychiatry. (2003) 42:386–405. 10.1097/01.CHI.0000046821.95464.CF 12649626

[12] Skerrett DM Barker E De Leo D. Suicide Research: Selected Readings, Vol. 8. A. I. for S. R. Prevention, Ed. (2012). Available online at: www.aapbooks.com

[13] Van Veen M Wierdsma AI Van Boeijen C Dekker J Zoeteman J Koekkoek B. Suicide risk, personality disorder and hospital admission after assessment by psychiatric emergency services. BMC Psychiatry. (2019) 19:1–8. 10.1186/s12888-019-2145-0 31122268 PMC6533743

[14] Slap G Goodman E Huang B. Adoption as a risk factor for attempted suicide during adolescence. Pediatrics. (2001) 108:1–8. 10.1542/peds.108.2.e30 11483840

[15] Zaborskis A Sirvyte D Zemaitiene N. Prevalence and familial predictors of suicidal behaviour among adolescents in Lithuania: a cross-sectional survey 2014. BMC Public Health. (2016) 16:1–15. 10.1186/s12889-016-3211-x 27405357 PMC4942925

[16] Valois RF Zullig KJ Hunter AA. Association between adolescent suicide ideation, suicide attempts and emotional self-efficacy. J Child Fam Stud. (2013) 24:237–48. 10.1007/s10826-013-9829-8

[17] Park E. The influencing factors on suicide attempt among adolescents in South Korea. J Korean Acad Nurs. (2008) 38:465–73. 10.4040/jkan.2008.38.3.465 18604156

[18] Office Office of Registrar General of India U. A Profile of Adolescents and Youth in India New Delhi (2014).

[19] Armstrong G Vijayakumar L. Suicide in India: a complex public health tragedy in need of a plan. Lancet Public Health. (2018) 3:e459–60. 10.1016/S2468-2667(18)30142-7 30219339

[20] National Crime Records Bureau. Accidental Deaths and Suicides in India, National Crime Records Bureau (Ministry of Home Affairs) Government of India, New Delhi (2018).

[21] India State-Level Disease Burden Initiative Suicide Collaborators. Gender differentials and state variations in suicide deaths in India: the Global Burden of Disease Study 1990 – 2016. Lancet Public Health. (2018) 3:478−89. 10.1016/S2468-2667(18)30138-5 30219340 PMC6178873

[22] Brent DA Perper JA Allman CJ. Alcohol, firearms, and suicide among youth: temporal trends in Allegheny County, Pennsylvania, 1960 to 1983. Jama. (1987) 257:3369–72. 10.1001/jama.1987.03390240075026 3586265

[23] Hill RM Castellanos D Pettit JW. Suicide-related behaviors and anxiety in children and adolescents: a review. Clin. Psychol. Rev. (2011) 31:1133–44. 10.1016/j.cpr.2011.07.008 21851804

[24] Cash SJ Bridge JA. Epidemiology of youth suicide and suicidal behavior. Curr. Opin. Pediatr. (2009) 21:613. 10.1097/MOP.0b013e32833063e1 19644372 PMC2885157

[25] Brent DA Mann JJ. Familial pathways to suicidal behavior — understanding and preventing suicide among adolescents. N Engl J Med. (2006) 355:2719–21. 10.1056/NEJMp068195 17192535

[26] Hawton K Van Heeringen K. Suicide. Lancet. (2009) 373:1372–81. 10.1016/S0140-6736(09)60372-X 19376453

[27] Orsolini L Latini R Pompili M Serafini G Volpe U Vellante F. Understanding the complex of suicide in depression: from research to clinics. Psychiatry Investig. (2020) 17:207. 10.30773/pi.2019.0171 32209966 PMC7113180

[28] Samuel D Sher L. Suicidal behavior in Indian adolescents suicide in India. Int J Adolesc Med Health. (2013) 25:207–12. 10.1515/ijamh-2013-0054 24006319

[29] Cole DA. Psychopathology of adolescent suicide: hopelessness, coping beliefs, and depression. J Abnorm Psychol. (1989) 98:248. 10.1037/0021-843X.98.3.248 2768660

[30] Rossetti MC Tosone A Stratta P Collazzoni A Santarelli V Guadagni E. Different roles of resilience in depressive patients with history of suicide attempt and no history of suicide attempt. Braz J Psychiat. (2017) 39:216–9. 10.1590/1516-4446-2016-2045 28538755 PMC7111383

[31] Chronis-tuscano A Molina BSG Pelham WE Applegate B Dahlke A Overmyer M. Very early predictors of adolescent depression and suicide attempts in children with attention-deficit/hyperactivity disorder. Arch Gen Psychiatry. (2010) 67:1044–51. 10.1001/archgenpsychiatry.2010.127 20921120 PMC3382065

[32] Hoertel N Franco S Wall MM Oquendo MA Kerridge BT Limosin F. Mental disorders and risk of suicide attempt: a national prospective study. Mol Psychiatry. (2015) 20:718–26. 10.1038/mp.2015.19 25980346

[33] Omigbodun O Dogra N Esan O Adedokun B. Prevalence and correlates of suicidal behaviour among adolescents in Southwest Nigeria. Int J Soc Psychiatry. (2008) 54:34–46. 10.1177/0020764007078360 18309757

[34] Kirkcaldy BD Eysenck W Siefen GR. Psychological and social predictors of suicidal ideation among young adolescents. Sch Psychol Int. (2004) 25:301–16. 10.1177/0143034304046903

[35] Klonsky ED May AM Saffer BY. Suicide, Suicide Attempts, and Suicidal Ideation (2016) 12:307–30. 10.1146/annurev-clinpsy-021815-093204 26772209

[36] Bridge JA Goldstein TR Brent DA. Adolescent suicide and suicidal behavior. J Child Psychol Psychiatry. (2006) 47:372–94. 10.1111/j.1469-7610.2006.01615.x 16492264

[37] Cho Y Haslam N. Suicidal ideation and distress among immigrant adolescents: the role of acculturation, life stress, and social support. J Youth Adolescence. (2010) 39:370–9. 10.1007/s10964-009-9415-y 20229228

[38] Jaen-varas D Mari JJ Asevedo E Borschmann R Diniz E Ziebold C. The association between adolescent suicide rates and socioeconomic indicators in Brazil: a 10-year retrospective ecological study. Braz J Psychiatry. (2019) 41:389–95. 10.1590/1516-4446-2018-0223 30785539 PMC6796813

[39] Jones JD Boyd RC Calkins ME Ahmed A Moore TM Barzilay R. Parent-adolescent agreement about adolescents' suicidal thoughts. Pediatrics. (2019) 143:245–55. 10.1542/peds.2018-1771 30642950 PMC6361346

[40] Pelkonen M Marttunen M. Child and adolescent suicide epidemiology, risk factors, and approaches to prevention. Pediatr Drugs. (2003) 5:243–65. 10.2165/00128072-200305040-00004 12662120

[41] Brown CR Hambleton IR Sobers-Grannum N Hercules SM Unwin N Nigel Harris E. Social determinants of depression and suicidal behaviour in the Caribbean: A systematic review. BMC Public Health. (2017) 17:1–1. 10.1186/s12889-017-4371-z 28619069 PMC5472962

[42] Sharma BR Gupta M Sharma AK Sharma S Gupta N Relhan N. Suicides in Northern India: comparison of trends and review of literature. J Forensic Leg Med. (2007) 14:318–26. 10.1016/j.jcfm.2006.08.009 17112767

[43] Santhya KG Acharya R Pandey N Singh SK Rampal S Zavier AJF. Understanding the Lives of Adolescents and Young Adults (UDAYA) in Bihar and Uttar Pradesh, India. New Delhi: Population Council (2017).

[44] Patel SK Santhya KG Haberland N. What shapes gender attitudes among adolescent girls and boys? Evidence from the UDAYA Longitudinal Study in India. PloS one. (2021) 16(3):e0248766. 33735285 10.1371/journal.pone.0248766 PMC7971892

[45] Kumar P Srivastava S Shankar P Sinha D. Children and Youth Services Review Does depressive symptoms, physical inactivity and substance use catalyze the suicidal tendency among adolescents ? Evidence from a cross-sectional study. Child Youth Serv Rev. (2020) 119:105661. 10.1016/j.childyouth.2020.105661

[46] Liu X Tein J Zhao Z Sandler IN. Suicidality and correlates among rural adolescents of China. J Adolescent Health. (2005) 37:443–51. 10.1016/j.jadohealth.2004.08.027 16310121

[47] Patton GC Olsson C Bond L Toumbourou JW Carlin JB Hemphill SA. Predicting female depression across puberty: a two-nation longitudinal study. J Am Acad Child Adolescent Psychiatry. (2008) 47:1424–32. 10.1097/CHI.0b013e3181886ebe 18978636 PMC2981098

[48] Dunlop SM More E Romer D. Where do youth learn about suicides on the Internet, and what influence does this have on suicidal ideation ? J Child Psychol Psychiatry. (2011) 52:1073–80. 10.1111/j.1469-7610.2011.02416.x 21658185

[49] Swanson SA Colman I. Association between exposure to suicide and suicidality outcomes in youth. CMAJ. (2013) 185:870–7. 10.1503/cmaj.121377 23695600 PMC3707992

[50] Ackard DM Neumark-sztainer D Story M Perry C. Parent–child connectedness and behavioral and emotional health among adolescents. Am J Prev Med. (2006) 30:59–66. 10.1016/j.amepre.2005.09.013 16414425

[51] Vajda J Steinbeck K. Factors associated with repeat suicide attempts among adolescents. Austral N Zeal J Psychiatry. (2000) 34:437–45. 10.1080/j.1440-1614.2000.00712.x 10881967

[52] Marttunen MJ Aro HM Lonnqvist JK. Adolescent suicide: endpoint of long-term difficulties. J Am Acad Child Adolesc Psychiatry. (1992) 31:649–54. 10.1097/00004583-199207000-00011 1644727

[53] Juan José López Ibor C Picazo-Zappino J. Suicide among children and adolescents: a review. Actas Esp Psiquiatr. (2014) 42:125–32. 24844812

[54] Shah SM Dhaheri F Al Albanna A Jaberi N Al Al S Alshehhi NA. Self-esteem and other risk factors for depressive symptoms among adolescents in United Arab Emirates. PLoS ONE. (2020) 15:e0227483. 10.1371/journal.pone.0227483 31935233 PMC6959560

[55] Soylu N Ayaz M Yüksel T. Early-married and sexually abused girls differ in their psychiatric outcomes. Child Abuse Negl. (2014) 38:1552–9. 10.1016/j.chiabu.2014.05.017 24994572

[56] Le Strat Y Dubertret C Le Foll B. Child marriage in the United States and its association with mental health in women. Pediatrics. (2011) 128:524–30. 10.1542/peds.2011-0961 21873691

[57] Zhu X Tian L Huebner ES. Trajectories of suicidal ideation from middle childhood to early adolescence: risk and protective factors. J Youth Adolesc. (2019) 48:1818–34. 10.1007/s10964-019-01087-y 31346925

[58] Christiansen E Jensen BF. Risk repetition of suicide attempt, suicide or all deaths after an episode of attempted suicide: a register-based survival analysis. Austral N Zeal J Psychiatry. (2007) 41:257–65. 10.1080/00048670601172749 17464707

